# DNR and COVID-19: The Ethical Dilemma and Suggested Solutions

**DOI:** 10.3389/fpubh.2021.560405

**Published:** 2021-05-12

**Authors:** Hala Sultan, Razan Mansour, Omar Shamieh, Amal Al-Tabba', Maysa Al-Hussaini

**Affiliations:** ^1^School of Medicine, University of Jordan, Amman, Jordan; ^2^Outcomes and Implementation Research Unit, Department of Internal Medicine, University of Kansas Medical Center, Kansas City, MO, United States; ^3^Department of Palliative Care, King Hussein Cancer Center, Amman, Jordan; ^4^Independent Researcher, Amman, Jordan; ^5^Human Research Protection Program, King Hussein Cancer Center, Amman, Jordan

**Keywords:** COVID-19, do not resuscitate, ethics, healthcare, Pandemic

## Abstract

Ethics are considered a basic aptitude in healthcare, and the capacity to handle ethical dilemmas in tough times calls for an adequate, responsible, and blame-free environment. While do-not-resuscitate (DNR) decisions are made in advance in certain medical situations, in particular in the setting of poor prognosis like in advanced oncology, the discussion of DNR in relation to acute medical conditions, the COVID-19 pandemic in this example, might impose ethical dilemmas to the patient and family, healthcare providers (HCPs) including physicians and nurses, and to the institution. The literature on DNR decisions in the more recent pandemics and outbreaks is scarce. DNR was only discussed amid the H1N1 influenza pandemic in 2009, with clear global recommendations. The unprecedented condition of the COVID-19 pandemic leaves healthcare systems worldwide confronting tough decisions. DNR has been implemented in some countries where the healthcare system is limited in capacity to admit, and thus intubating and resuscitating patients when needed is jeopardized. Some countries were forced to adopt a unilateral DNR policy for certain patient groups. Younger age was used as a discriminator in some, while general medical condition with anticipated good outcome was used in others. The ethical challenge of how to balance patient autonomy vs. beneficence, equality vs. equity, is a pressing concern. In the current difficult situation, when cases top 100 million globally and the death toll surges past 2.7 million, difficult decisions are to be made. Societal rather than individual benefits might prevail. Pre-hospital triaging of cases, engagement of other sectors including mental health specialists and religious scholars to support patients, families, and HCPs in the frontline might help in addressing the psychological stress these groups might encounter in addressing DNR in the current situation.

## Introduction

Cardiopulmonary resuscitation(CPR) was first devised in 1530 (1). However, it was only in 1956 that CPR was reinvented and refined into the currently known and performed technique (2). Do-not-resuscitate (DNR) is defined when neither basic (heart compressions and ventilation) nor advanced (defibrillator or medicines) CPR should be performed. Terminally ill patients for which further medical intervention is considered futile, when quality of life is deemed poor, or who are expected to be permanently dependent on ventilators are the cases in which DNR is considered a plausible decision. The DNR decision is usually made based on a combination of themedical decisions and the patient's wishes and values. Interestingly though, the legal status of DNR varies between countries; from allowing it to a complete prohibition with legal consequences (personal communication). A link between ethics and DNR became a heated topic and the subject of published literature in 1979 (3), and later on addressed in further publications (4–8). Many linked religious and psychosocial conditions (9), and spirituality (10), as well as ethnicity (11) to the acceptance of the DNR order. Other features like chronic illness and old age may also impact the DNR decision (12–14), although a patient as young as 40 years old might succumb to the DNR order in the face of certain medical conditions (15). Children with DNR orders serve yet another example where physicians might encounter hardship as parents/ guardians have to make difficult decisions (9, 16). Also, more acute conditions within the setting of an intensive care unit (ICU) for example can elicit a DNR order (17). In the setting of lower-respiratory tract infection/pneumonia for example, DNR orders resulted in lower hospitalization and hospital-based mortality incidences suggesting that even in the absence of outbreaks and pandemics, planning and implementing DNR would save resources which can then be re-directed (18, 19).

Resources can become especially scarce during a pandemic. The World Health Organization (WHO) defines a pandemic as “the worldwide spread of a new disease” (20) where the R0, a term that reflects “how infectious a disease is,” is >1 (21). In the more recent era, the world had witnessed many disease outbreaks, some of which were declared worldwide pandemics. These include, but are not limited to, the Asian flu in 1957, Severe acute respiratory syndrome (SARS) in 2002, Ebola in 2014, and lastly Zika in 2015 (22).

A thorough search affirmed that the closest recent pandemic to the current COVID-19 pandemic is H1N1 influenza (R0 = 1.4 −1.6). Back in 2009, the UK's Resuscitation Council established guidelines regarding CPR and the H1N1 influenza pandemic (22). It affirmed that DNR patients should be identified early on so that no CPR is attempted. However, in the case of commencement with CPR, only chest compressions should be started; mouth-to-mouth ventilation should be avoided. Recently, an article on the American Heart Associations' guidance for CPR amid the COVID-19 pandemic reiterated the aforementioned H1N1 guidelines, and also emphasized the use of airborne infection isolation rooms especially when there is a risk of dissemination of virus droplets, such as endoscopies, and bronchoscopy procedures, as well as respiratory protection; most importantly an N95 mask (23). More importantly, physicians are recommended to intubate patients with respiratory failure owing to the COVID-19 virus to reduce the risk of aerosol generation (24). The current pandemic, owing to the pervasive COVID-19 virus, with up to 100 million cases worldwide and 2,170,000 deaths (January 27, 2021), advocates for upfront implementation of the DNR order to COVID-19 infected patients; especially the elderly or those deemed associated with poor prognosis as per the physician's assessment (25). The inquiry here is multifaceted. How ethical is it to consider unilateral (i.e., without prior consent of patient) DNR orders for COVID-19 infected patients in the face of limited resources? What are the potential consequences for other patients suffering from acute heart conditions, respiratory conditions, or road traffic accidents who might be competing with COVID-19 infected patients for the limited ventilators? Can we deny pre-planned treatment management to certain groups of patients (like for example new and on-treatment cancer patients) to preserve needed ICU rooms and ventilators if unilateral DNR orders for COVID-19 infected cases could not be made? And essentially, what are the moral consequences for the healthcare providers (HCPs) making these tough decisions? How might these measures interject with the four major principles of medical ethics; autonomy, beneficence, non-maleficence, and justice? What would be a plausible approach to this ethical dilemma?

To address these questions, an exhaustive literature review using PubMed, Medline, Science Direct, and online news sites was undertaken to gather evidence and summarize the local, regional, and international recommendations.

How ethical is it to consider unilateral DNR orders for COVID-19 patients in the face of limited resources?

The answer to this question might not be straight forward. Given COVID-19's very high R0 as well as the relatively low success rate of CPR among ICU patients in general and the scarcity of personal protective equipment (PPE), many “hospitals on the frontline of the pandemic are attempting to weigh the costs of exposing doctors and nurses to the coronavirus” (26). On a global level, the issue is; what happens if HCPs who are at the frontline in our battle against COVID-19 get infected with the virus in an attempt to resuscitate a patient with a very low probability of survival, i.e., older people with preexisting comorbidities; including cardiac, respiratory, and other chronic health disorders (27)? It is also important to recall that the possibility of discharging an ICU patient after using CPR is around 17% (28), and that CPR is only effective in the first 4 to 7 min of cardiopulmonary arrest; by the time physicians reach the patient, especially if they had to wear PPE, they might have already run out of time. On the other hand, rushing to respond to CPR situations can increase the probability of PPE breach, putting HCPs at risk of infection (26). Moreover, most patients who are successfully resuscitated will need a ventilator, further contributing to the scarcity of resources amidst the COVID-19 pandemic, and possibly depriving other patients with a greater probability of survival from using these resources. In light of these debatable questions, DNR seems to be an immensely valid option.

An article by Curtis et al. (27), “Decisions About Do-Not-Resuscitate Orders During COVID-19,” emphasizes the dangers of COVID-19 and that its spread has led to the development of so-called “unilateral DNR.” This term was coined to “reduce the risk of medically futile CPR to patients, families, and healthcare workers.” This is especially when CPR will unlikely allow successful return to an acceptable quality of life. It also saves ICU resources to allow for the accommodation of patients with a better chance of recovery. In the case that such protocols are implemented, all patients and family members should be knowledgeable about and adhere to the healthcare unit's wishes.

One such example is in New Jersey and some hospitals in New York; as of March 27, 2020, all “COVID-19 patients [will be placed] on a DNR-B resuscitation status.” In DNR-B, all patients continue to receive their treatment for all medical conditions except in the event of a cardiac arrest (29), “No code blue will be called on any COVID-19 patients.” (30) Other states in the United States, as well as other countries, are yet to decide.

A study in 2016 that addressed the ethicality of allocating scarce medical resources by HCPs explored the views of general practitioner (GP), medical students, and lay people (31). In one of the scenarios addressed (Scenario-B), allocation of scarce beds in hospitals amid an imaginary flu epidemic, lay personnel ranked the “sickest” patient as the priority in the limited bed allocation, while “prognosis” was top rated by the GP, medical students, and other HCPs. This clearly addressed the potential controversy that might arise among HCPs and patients during health crises like pandemics and that should be addressed in anticipation of any.

Whether patients infected with COVID-19 can be considered as a vulnerable population (people in need of special care, support, or protection because of age, disability, or risk of abuse or neglect) warrants further consideration while addressing the issue of DNR. Age was among the discriminator to triage patients; patients older than 80 years were offered DNR because of the futility of treatment and co-morbidities (25). Patients and families of patients diagnosed with COVID-19 disease have been stigmatized in some communities, which further adds to the vulnerability of COVID-19 patients (personal communication).

What are the potential consequences for other patients suffering from acute heart conditions, respiratory conditions, or road traffic accidents who might be competing with COVID-19 patients for the limited resources?

Triaging patients including COVID-19 patients, those with acute conditions like cardiopulmonary cases, those with emergency surgical intervention as well as cancer patients planned for elective surgeries which can be postponed for a maximum of a few weeks, but no longer, would be an important ethical consideration when addressing the potential of limited resources should ICUs and ventilators be needed. In Italy, around 50% of hospital beds in a 1,000-bed hospital in Northern Italy were occupied by COVID-19 patients (32), leaving the other half to deal with the rest of the other medical conditions, which might be sub-optimal to say the least. As a consequence, elective surgeries have been canceled, semi-elective procedures postponed, and operating rooms turned into makeshift ICUs (32).

The practice of dealing with DNR is sub-optimal even in the luxury of the routine practice outside pandemics. Within the setting of oncology practice in particular, Pettersson et al. reported that almost half of the nurses and physicians surveyed on the issue of DNR reported that “it is not likely that the patient would be involved in the decision on DNR,” 21% believed that it is irrelevant to inform patients of the DNR decision, and 57% reported that providing information to the patient was important, although only 21% stated that this was likely to happen (33). Importantly, Bovman argues that reversing a DNR code if elective surgery is warranted is associated with a dismal 30-day mortality (34). One important limitation is that patients treated for other conditions might end up infected with COVID-19 once admitted to the hospital (32).

At King Hussein Cancer Center (KHCC), the only stand-alone cancer center in Jordan, difficult decisions had to be made as well (35). During the months of March, April, and May, all elective surgeries, clinical appointments, and procedures were canceled, and chemotherapy and radiotherapy were canceled for the first two weeks and then started to build up gradually during the third week in anticipation of a potential surge of COVID-19 infected cases. In addition, patients were instructed to call a designated hotline if needed instead of in-person arrival to KHCC. A fully prepared ward was assigned to quarantine confirmed COVID-19 patients. All non-frontline employees were asked to stay at home, and a minimal number of HCPs were scheduled to cover the needs. Although this could compromise the small windows cancer patients might have, difficult decisions are made in anticipation of the worst (Ethical Considerations for Treating Cancer Patients during the SARS-COV-2 Virus Crisis: To Treat or Not to Treat? A Cancer Center in Low-Middle Income Country).

### What Are the Consequences for HCPs Taking These Tough Decisions?

In harmony with the Hippocratic Oath, every medical physician swears to “apply, for the benefit of the sick, all measures [that] are required” (36). This regards not only to day-to-day practice, but also, and more importantly, for when they are needed most, such as in times of outbreaks. Accordingly, the consequences for the HCPs can be divided into physical/physiological and psychological/moral injuries.

To HCPs, and in accordance with what their degrees encompass, universal DNR to COVID-19 infected patients does not seem to be an option, adding to the ethical dilemma, self-blame, and burnout of the frontline decision makers. In some countries like the US, hospitals should apply for a so-called 1135 waiver, that waiver temporarily lifts Centers for Medicare & Medicaid Services requirements in times of a national emergency, because failing to do so is considered “in violation of patient rights”(37). In a study that addressed frontline vs. non-frontline nurses dealing with COVID-19 patients, a significant difference in both physiology and psychology between both groups was in favor of frontline nurses (38).

As for the psychological/moral consequences, in this particular setting of the COVID-19 pandemic, initiating or terminating a life supporting ventilator might be among the most difficult acts a physician can make during his/her career (39). Italian physicians were reported to weep in hospital hallways because of the difficult decisions they had to make (40).

It would be of interest to investigate if the HCPs in the current pandemic understand the burden of approving a DNR for patients infected by COVID-19. While this might be unreachable in the current condition, to understand the impact of the one-way tough decisions made by the physicians should be the subject of further research.

### How Might These Measures Interject With the Four Major Principles of Medical Ethics; Autonomy, Beneficence, Non-maleficence, and Justice?

The dispute here is whether DNR codes, especially the unilateral DNR code, and resuscitation guidelines respect the four core medical ethics principles: autonomy, beneficence, non-maleficence, and justice (41).

### Autonomy

Autonomy and non-maleficence were reported by nurses and physicians, respectively, as the most important ethical values when dealing with the DNR status (42). Deciding on DNR on behalf of patients, i.e., unilateral DNR to save others with a higher probability of survival and to protect HCPs may serve the principles of equity and not equality, and seems to violate the principle of autonomy, which honors the patients' preference and wishes regarding any decision for their medical care. Fostering autonomy would dictate the discussion of all care-related options including the DNR code and do-not-operate (DNO) code with the patient and/or family so that they can make an informed decision (43–45). An informed consent form signed by the patient or a surrogate might, however, falsely re-assure the HCPs of the patient's understanding and thus volunteerism and autonomy (46). Also, making decisions on behalf of a competent patient exemplifies a paternalistic and professional nihilism that contradicts autonomy (47). Additionally, weighing the risk-to-benefit ratio and prioritizing societal over individual benefit is another issue when considering DNR, especially amid the COVID-19 pandemic.

### Justice

The principle of justice entails “fair adjudication between conflicting claims,” as well as treating patients with fairness, and to do so equally and equitably (48). Concerning the COVID-19 pandemic and DNR, the term “distributive justice” resurfaces, which considers fair allocation of resources, treatments, and benefits during a time of medical resource scarcity. Physicians started treating patients equitably but not equally, and other factors entered the equation when it came to providing care, as patients with the best chance of recovery were prioritized over others (49). Moreover, due to prolonged exposure, close contact, and lack of PPE, healthcare workers are at a significantly increased risk of acquiring infection (50), and should be prioritized when providing critical care when it comes to advanced life support.

What medical and ethical decision should be made when all patients are equal in need and predicted outcome, but the resources are barely enough? One study proposed a central “lottery” system as a solution for the distribution of resources to these patients. Patients' characteristics were suggested to be entered into the system and a supervised random selection process should then take place to ensure fair and equity of distribution (51). This could also apply to patients who are predicted to need CPR. However, the controversy will still be an issue, and there will be no single “best” answer.

### Beneficence and Non-maleficence

Beneficence is defined as “an act of charity, mercy, and kindness with a strong connotation of doing good to others including moral obligation” (52). In healthcare, beneficence encompasses the idea that a physician's actions, decisions, and skills must always advocate for what is best for the patient. Physicians must apply the principle of beneficence while causing no harm to patients, a term referred to as non-maleficence (“above all do no harm”). In this instance, CPR is advised to be performed on patients if apparent benefit was the expected result. However, some argue that CPR should not be performed if it is not expected to result in benefit to patients, or if it may prolong their suffering, and the physicians should accordingly write a unilateral DNR order (53). The ethical and medical decision depends upon weighing therapeutic benefits against risks.

### What Would Be a Plausible Approach to the Ethical Dilemma?

**Figure 1:**
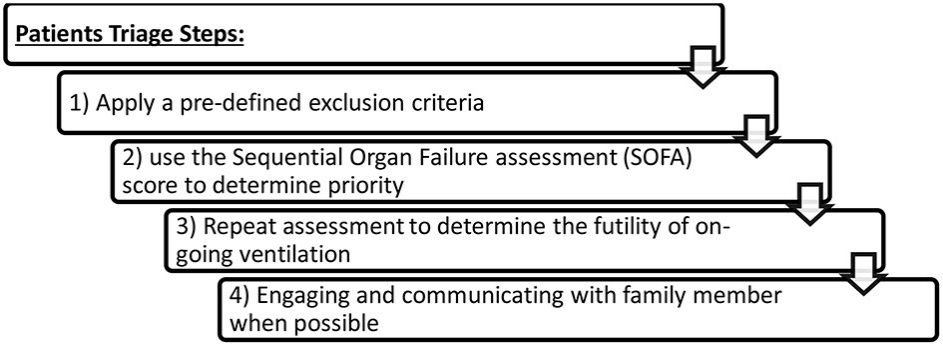
Patient triagesteps.

In a more conscious evaluation of the objective indications of DNR, Lipsky identified four core elements that can be assessed when deciding on DNR; futility of treatment, poor quality of life, patient refusal, and cost (54). If these same elements are applied into the current condition, where societal benefit prevails over self-benefit, it would be logical to consider any of the aforementioned four elements as a justification for the universal or unilateral DNR code adopted by the health sector in some nations. Along the same lines, Edwards B.S., argues that a small but significant number of ICU DNR-coded patients consume the already scarce resources including HCPs; nurses in this particular case within normal circumstances (55), let alone the current COVID-19 pandemic and the strain on limited resources. Calls for a just allocation for the use of the already limited resources are in place despite potential adverse effects on patient's autonomy and beneficence. Additionally, an important argument would be that an early DNR code would save the patient and family futile interventions (13). Triaging patients can be a multi-step and dynamic process that consists of three steps including (1) the application of exclusion criteria, (2) using the Sequential Organ Failure Assessment (SOFA) score to determine priority, and (3) repeated assessments to determine the futility of on-going ventilation (39). We would suggest a fourth point for engaging and communicating with family members when possible (Figure 1).

**Figure 2:**
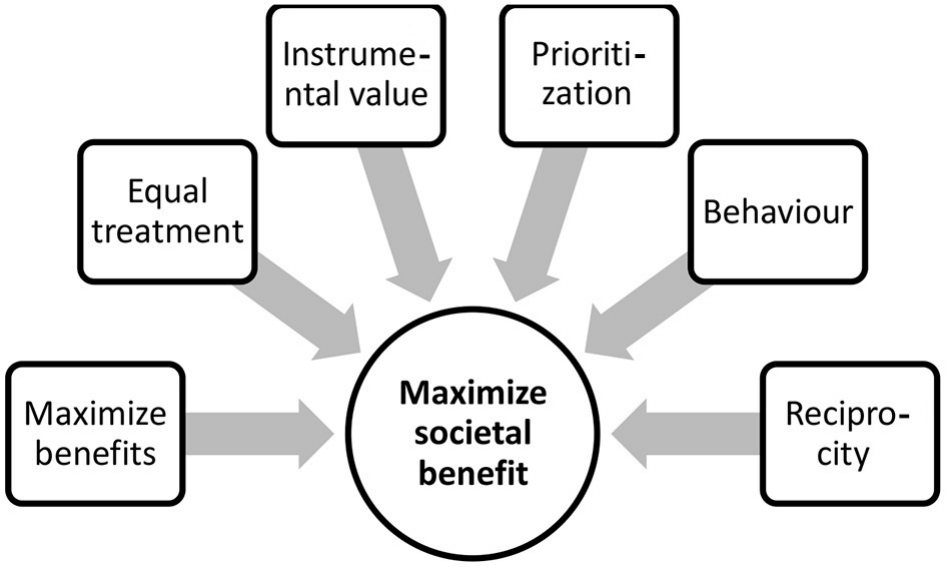
A multi-valueethical framework to maximize societal benefit.

Curtis et al. shared an algorithm on how to address advanced care planning, the goals of care, and informed assent with a patient or surrogate family. This should proceed stepwise so that the patient and/or family surrogate can affirm understanding or are otherwise allowed to object (27). Since not one single ethical consideration might be able to address how to allocate scarce resources, a multi-value ethical framework, where more than one factor is considered, might seem more ethical (25). Maximizing benefits, i.e., saving the most lives and treating those with better prognosis, equal treatment to people, i.e., selection among people with similar prognosis, instrumental value, i.e., benefits to others, and priority to the sickest or the youngest when it aligns with maximizing benefits, should all be combined to maximize societal benefit. Additional factors that we suggest based on this literature review to help align scarce resources include behavioral status; priority to those who did not engage in risky behaviors that caused their condition or affected it negatively, and reciprocity; priority to those who have voluntarily provided societal services in the past (Figure 2).

Deployment of the medical workforce in areas in most need is an effective modality to support healthcare systems. This has been an effective strategy in Wuhan, China, where attempts to contain the spread of the pandemic was a wise decision (38). In the US, due to the likelihood of a shortage of HCPs, many retired physicians and medical students volunteered to aid in the crisis. Dr. Judy Salerno, a retired physician in her 60s declared that “if (she) can use (her) skills in some way that will be helpful, (she) will step up” (56). Medical students have also aided, taking basic histories over phone calls and babysitting for HCPs overwhelmed in hospitals and other facilities. In Jordan, medical students were heavily engaged with surveillance activity for potentially infected persons, as well as volunteering to deliver prescribed drugs to patients (personal communications).

The psychological impact on HCPs is of paramount importance (57), and should be accounted for when nationwide decisions are put in place (38). It is of value to note that whether or not a physician or hospital desires to commence with the unilateral DNR protocol, this decision should not be left entirely up to them; “providers, administrators, attorneys, clergy, and compliance” should be called to discuss the specifics (20). Proper training and education of the HCPs, especially junior staff, should be in place to help alleviate misconception on the timing of, and inclusion/exclusion criteria of the DNR code (58, 59). In addition, DNR needs to be disclosed by the more experienced members in the caring team (60). Many hospitals would triage CPR/DNR patients in the hand of a committee, none of the members of which are involved in patient treatment (39). One suggestion is to create a “triage committee” composed of senior and respected members of the medical community who volunteer to sit in on these committees, thus preventing first-line HCPs from making tough decisions that may impact their well-being. KHCC has adopted a similar approach, where the decision on unilateral DNR has to be made by a committee composed of the primary physician and two other physicians for terminally ill patients if a shortage in ventilators occurs in the future in Jordan (35).

An often overlooked facet is the role religious scholars can play when a DNR order is made. Religious scholars for different theistic groups should be made part of the clinical ethics committees in the hospitals, and in the case of the COVID-19 pandemic, national committees that address the DNR issue in acutely diseased and admitted infected patients (61). Providing support to the patient and/or family should also be extended after discussing the DNR. The presence of ethics-trained religious scholars can be of utmost importance especially when confronting national crises to ensure patient dignity, coping strategies for the family, and relief of the HCPs, with an ultimate goal to support family members as well as HCPs. Of interest, the European Islamic Jurisdiction Council clearly addressed the social impact on larger communities associated with the COVID-19 pandemic. Driven by the larger societal benefit, DNR orders were endorsed if deemed necessary by a compatible physician (62).

## Conclusion

Despite the ethicality of this matter, and as a result of the rapid evolution and progress of the COVID-19 pandemic, as well as the anticipated shortage of resources, some hospitals have already made decisions. Public trust and confidence in the medical decision should not, however, be overlooked. Transparency of the medical sector, along with public engagement should help in alleviating the ethical burden of applying the unilateral DNR to COVID-19 infected patients and maintaining public trust. Practical approaches are suggested to address the potential sequelae. All in all, the question facing HCPs here may not precisely be how ethical, but rather: what choice do you make when 7.8 billion people's lives are at risk?

## Author Contributions

HS: literaturereview, writing the first draft, and review and final approval. RM: literature review, reviewing the first draft, and final review and approval. OS: literature review, critical review of the draft manuscript, and final review and approval. AA-T: literature review, reviewing the first draft, and final review and approval. MA-H: inception of the idea, literature review, critical review of the first draft, and final review and approval. All authors contributed to the article and approved the submitted version.

## Conflict of Interest

The authors declare that the research was conducted in the absence of any commercial or financial relationships that could be construed as a potential conflict of interest.
